# Supplementary material for: Development of a computer-aided design software for the quantitative evaluation of aesthetic damage
Source: PLoS One. 2019 Dec 18;14(12):e0226322. doi: 10.1371/journal.pone.0226322 (PMC6919621; doi:10.1371/journal.pone.0226322)
Supplement: S1 Code site — 1. (ZIP) [file pone.0226322.s001.zip › [EESC_jr][Projeto]Relatorio_07_21/reports/templates_zip/gentelella-master/vendors/echarts/test/dataZoom-axis-type.html]

chart.setOption({
dataZoom: [
{
id: 'xSlider',
startValue: '类目36',
endValue: '类目90'
}
]
});
chartIndex:

chart.setOption({
dataZoom: [
{
id: 'xSlider',
startValue: '2011-02-04',
endValue: '2011-05-06'
}
]
});
chartIndex:
